# Higher accumulation of mitragynine in *Mitragyna speciosa* (kratom) leaves affected by insect attack

**DOI:** 10.1371/journal.pone.0320941

**Published:** 2025-04-29

**Authors:** Nisa Leksungnoen, Tushar Andriyas, Rossarin Tansawat, Pichaya Pongchaidacha, Piyawadi Khaoiam, Thanundorn Thanusuwannasak, Chatchai Ngernsaengsaruay, Chawatat Thanoosing, Suwimon Uthairatsamee

**Affiliations:** 1 Department of Forest Biology, Faculty of Forestry, Kasetsart University, Bangkok, Thailand; 2 Department of Food and Pharmaceutical Chemistry, Faculty of Pharmaceutical Science, Chulalongkorn University, Bangkok, Thailand; 3 Metabolomics for Life Sciences Research Unit, Chulalongkorn University, Bangkok, Thailand; 4 Chulalongkorn University Drug and Health Products Innovation & Promotion Center, Faculty of Pharmaceutical Sciences, Chulalongkorn University, Bangkok, Thailand; 5 Department of Botany, Faculty of Science, Kasetsart University, Bangkok, Thailand; 6 Department of Biology, Faculty of Science, Chulalongkorn University, Bangkok, Thailand; Qingdao Agricultural University, CHINA

## Abstract

Kratom leaves are widely consumed by locals to increase their stamina for working longer hours in the field. However, insect damage to the leaves can lead to significant loss of leaf harvest. Despite this, there remains considerable uncertainty regarding how herbivory attack affects the chemical composition of kratom’s leaf metabolome. In this study, we investigated the effect of insect herbivory on the secondary metabolites (SMs) of kratom leaves, using untargeted metabolomics as well as the effect on the accumulation of mitragynine and relation to leaf traits. The presence of diverse herbivore species were observed on the kratom leaves as indicated by three orders of insects including Coleoptera (beetles), Lepidoptera (butterflies and moths) and Hemiptera (true bug). A higher accumulation of mitragynine was observed as a defense mechanism against herbivores, with the study also finding a correspondence between increased mitragynine levels and elevated leaf acidity. A significant difference in the presence of three key metabolites (cearoin, 8-hydroxy-8-(3-octyloxiran-2-yl) octanoic acid, and 2,3,5,7-tetramethoxy-9,10-dihydrophenanthrene) that have interesting therapeutic usage, between insect and non-insect leaves, indicated the influence of interaction with insect herbivory. In conclusion, these findings highlight the potential to manage insect herbivory to modulate alkaloid accumulation in kratom, offering a sustainable approach that preserves insect biodiversity while naturally boosting the plant’s chemical defense.

## Introduction

Higher plants synthesize a wide range of secondary metabolites (SMs) to protect or survive biotic and abiotic stresses. These include alkaloids, anthocyanins, flavonoids, quinones, lignans, steroids, and terpenoids, which are used as pharmaceuticals, agrochemicals, flavors, fragrances, colors, biopesticides, and food additives [1]. Spatial and temporal variations in the levels of SMs create a potential multidimensional feeding ecosystem [2]. It has been previously reported that the presence and variations in nutrients and SMs can influence foraging [3,4], with certain SMs reducing the loss of biomass due to herbivory without inducing acute poisoning or death [5].

Moreover, SMs tend to limit the amount of ingested toxic food, indicating that presence of SMs is not only an avoidance but a regulation mechanism as well [6–8]. SMs like tannins block nutrient utilization [9], while terpenoids and other compounds can cause a reduced intake of the food high in energy. Specialist insects have evolved various strategies to use SMs to improve their fitness, as in the sequestration of pyrrolizidine alkaloids from the host plant by the arctiid moth *Utetheisa ornatrix*, to deter predators and attract potential mates [10]. Additionally, therapeutic self-medication through the preferential consumption of SM-rich plants by herbivores has been reported to bolster their health and immunity against pathogens [11]. Furthermore, detoxification through liver enzymes and gut microbiota can help herbivores to metabolize and tolerate toxic SMs, thus offsetting potential fitness costs associated with herbivory [12]. The presence and selective consumption of kratom leaves by insects presented in the present study might be used to exploit its unique foliar chemical profile for survival and reproduction. Exogenous factors like herbivory can trigger intrinsic defense mechanism involving SMs as deterrents.

The functional diversity of SMs ranges from resistance to abiotic stresses (e.g., UV radiation, drought, heat) to modulating interactions with antagonists (e.g., competing species, pathogens, herbivores) and mutualists (e.g., mycorrhizal fungi, pollinators, rhizobium bacteria, predators/parasitoids of herbivores) [13]. Although such SMs do not influence the maintenance of fundamental life processes, they mediate the interaction of the plant with its environment and also act as defense chemicals [14]. The production of these compounds is very low (less than 1% dry weight), depending mainly on the physiological and developmental stage of the plant [6,15]. Interactions between plants and herbivores or pathogens are driven through production, allocation, accumulation, and diversity of SMs among various plant tissues, so as to deter [16,17] or attract [18] herbivores. Several previous studies have investigated the interplay between plant chemical traits and herbivore performance in terms of chemical compounds, including alkaloids, phenolics, flavonoids, nonprotein amino acids, or glucosinolates [19–22].

Typically, the production of SMs is relatively low but their biosynthesis increases tolerance to environmental stimuli. The accumulation of such compounds can be elevated to significant levels in plants under herbivore attack, as reported by up to 6% usage of *Nicotiana attenuata* plant nitrogen pool to the production of the alkaloid nicotine [23]. In the cultivation and field experiments, leaf beetle consumed leaves with elevated levels of condensed tannins and total phenolics in *Rumex obtusifolius* plants relative to leaves in other treatments [24]. It has been recently reported that wheat genotypes with moderate resistance to cereal leaf beetle had higher production of flavonoids [25]. Such elevated accumulation of SMs is usually causated by significant changes in primary metabolism after an insect or pathogen attack [26–28].

Several secondary metabolites (SMs), particularly alkaloids, share structural similarities with neurotransmitters and have evolved as either agonists or antagonists of nervous signal transduction, impacting ionic pathways, neurotransmitter receptors, and transporters [29]. Such modulation can significantly alter insect physiology and behavior. Alkaloids, one of the largest classes of SMs, are found in approximately 20% of known vascular plants and are classified into several groups like quinolizidine, indole, pyrrolizidine, and tropane alkaloids [30,31]. These compounds have been reported to exhibit notable anti-herbivore and anti-microbial properties [32,33]. For instance, elevated levels of solanidine in *Solanum* species is linked to reduce leafhopper infestations [34], while the foliar alkaloid gramine in barley can influence the resistance to the aphid *Schizaphis graminum* [35]. Additionally, *Festuca arundinacea* colonized by the fungal endophyte *Acremonium coenophialum* tends to have a reduction in aphid feeding due to increased pyrrolizidine alkaloid levels, which are toxic to various insects [36].

Plants usually respond to herbivory by upregulating the production of specific SMs, increasing the production efficiency of these metabolites, which can reduce the likelihood of subsequent herbivore attacks [37]. However, the effectiveness and nature of this response can vary depending on the species of herbivore, its specialization, and its feeding guild [38]. Some SMs are expressed selectively, particularly in environments visited by frequent herbivory, offering plants a consistent level of defense [39]. A notable alkaloid found in kratom is mitragynine, which is the principal bioactive compound identified in the plant [40] and known for its pharmacological properties.

Insect feeding on kratom is an issue that can reduce leaf quality, while response to such stress might lead to an increased production of SMs like mitragynine. Understanding this interaction is crucial for developing insect management strategies that can enhance alkaloid content while reducing pesticide use. This study explores how interactions with herbivory affects the presence of SMs and the accumulation of mitragynine in kratom in mounting a chemical defense. This knowledge will be beneficial for farmer who would like to control insect harmfulness and also to increase the concentration of chemical in kratom regarding to the interaction between insect and SMs production, improving both crop quality and sustainability.

## Materials and methods

### Study site

**Fig 1:**
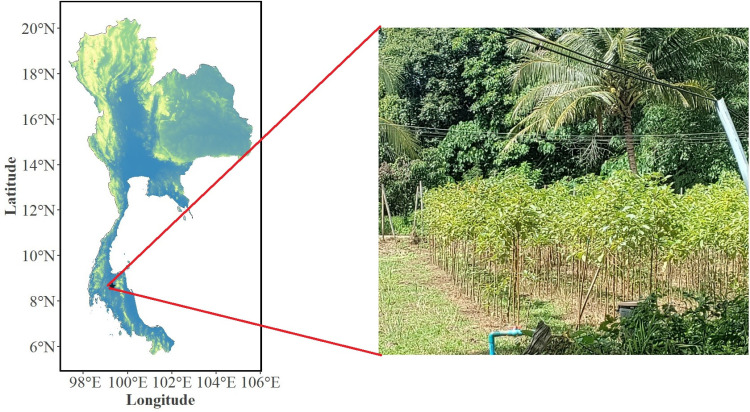
Kratom field located in the southern part of Thailand from which insect and non-insect leaves were sampled. The map of Thailand was generated using R statistical software [42] with the raster colors indicating the mean elevation above sea level. The map was generated using publicly available data and open-source R packages (suitable for use under the CC BY 4.0 license). The world administrative boundaries shapefile was obtained from https://public.opendatasoft.com/explore/dataset/world-administrative-boundaries/export/ and imported into R software using the *st_read* function from the *sf* package. The digital elevation map (DEM) for Thailand was accessed using *getData* function of the *raster* package and cropped to the country of Thailand using the *crop* function of the *terra* package, while the final visualization was done using the *ggplot2* package.

Experiment was conducted in the southern part of Thailand on a 1.5 year old monoculture kratom plantation (in an area of around 0.8 hectare), located in Namphu, Ban Nasan, Surat Thani province (8.764545N, 99.301739E) (Fig 1), with narrow spacing of 50 cm. × 150 cm, owned by Mr. Suphawat Klomwiset, a farmer and citizen scientist. The climate is hot and humid with at least eight months of rainfall (more than 3000 mm annually). The air temperature usually ranges between 22–33^0^C with a very low vapor pressure deficit (less than 1.0 kPa), with the light intensity usually remaining high all year round between 16–21 MJ m^-1^day^-1^ [41]. The kratom plantation was irrigated two times a day using a sprinkler until the soil was saturated to mimic swamp like conditions observed in its native habitat.

### Insect diversity

#### Insect sampling.

The insect sampling was conducted on January (13^th^), during March (26^th^–28^th^), and June (15^th^ –16^th^), 2024 using hand nets and forceps during the daytime (from 1 PM to 4 PM). The insect collection procedure followed the guidelines outlined by the Thailand Institute Animal Care and Use Committee (Thai IACUC) under the License ID: U1-9787–2565. Light traps were also used to collect insects at night in the month of March and June (from 7 PM to 10 PM). The target insects for the light traps were adult moths, collected as reference for pairing with juveniles found during the daytime. The potential insects were collected in the whole plantation (containing around 1400 trees), recorded and photographed by the co-author and insect specialist (CT). The insect samples were preserved in 95% ethanol to prevent any molecular degeneration. The samples were kept at -20˚C at the Bee and Spider Research Unit, Chulalongkorn University, Bangkok.

#### Insect identification via molecular techniques.

The samples were sorted into respective morphospecies under a stereomicroscope ZEISS SterREO Discovery V8. Subsequently, one or two representative samples of each morphospecies were selected for DNA extraction. The images of representatives were taken using a stereomicroscope ZEISS Stermi 508 with a Canon DSLR EOS 90D. The genomic DNA of the sample was extracted using the TIANamp Genomic DNA Kit (TianGen, China), following the kit protocol. The DNA concentration was checked using NanoPhotometer version NP80 (IMPLEN). The cytochrome c oxidase subunit I gene or COI, as the animal DNA barcode for identification, was amplified using Polymerase chain reaction (PCR) with OneUltraPCR supermix (GeneDirex, Inc.). The primers used for the PCR were LepF1 5’-ATTCAACCA ATCATAAAGATAT-3’ and LepR1 5’-TAAACTTCTGGATGTCCAAAAA-3’ [43]. The PCR temperature profile was one cycle of pre-denaturation at 95°C for 2 minutes; 40 cycles of denaturation at 94°C for 40 seconds, annealing at 52°C for 40 seconds, and extension at 95°C for 2 minutes; and single cycle of final extension at 72°C for 5 minutes. The PCR products were examined for their size using 1.5% agarose gel electrophoresis at 70 volts for 30 minutes with a 1kb DNA ladder. The gels were checked under a UV transilluminator. The PCR products were purified using the TIANquick Midi Purification Kit (TianGen, China). The purified PCR products were sequenced in the forward direction using Sanger sequencing techniques at the ATGC company ltd., Thailand (https://atgc.co.th/). The COI sequences were checked and edited, according to electropherogram, using MEGA11 [44]. Any unreliable parts, including the beginning and end of each sequence, were discarded. The reliable sequences were compared with GenBank database using BLAST (Basic Local Alignment Search Tool) [45]. The voucher specimens were deposited to the Chulalongkorn University Museum of Natural History as dry pinnings for adult insects, and wet preservation for juvenile insects.

### Leaf measurements and collection

Mitragynine is recognized as the primary alkaloid in kratom leaves, and its accumulation was quantified in insect-damaged leaves relative to undamaged ones. The underlying hypothesis was that biotic stress resulting from insect damage would stimulate an increase in alkaloid production as a defensive response. This increase was theorized to manifest as increase in the bitterness of the leaves, thereby deterring further insect herbivory. For the analysis, mitragynine was selected due to its predominance within the alkaloids found in kratom leaves [40]. To control for potential confounding variables, such as leaf age, ten biological replicates from leaf samples were collected from leaf pairs on the same plant. This approach minimized any age-related variations and provided a more accurate comparison of alkaloid levels between damaged and undamaged leaves.

**Fig 2:**
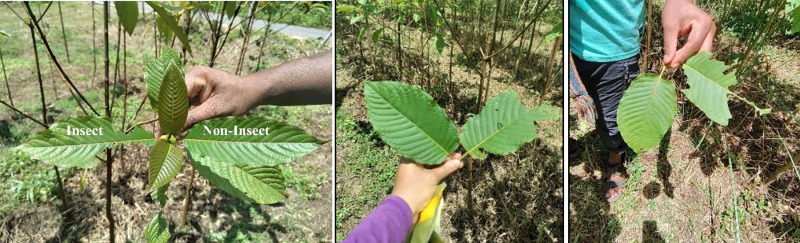
Visual observations of insect attack on kratom leaves in the plantation in Nampu subdistrict, Ban Na San district, Surat Thani province.

The leaf arrangement of kratom is decussate with the same node containing two leaves of the same age but growing in opposite direction. We collected only the second and third leaf pair at the same node, with one leaf damaged by the insect while the other leaf being undamaged (Fig 2). Fifty replicates of both types of leaves (damaged and undamaged) were randomly collected throughout the field. The physiological characteristics were measured including leaf thickness, specific leaf area, chlorophyll content, maximum quantum yield, performance index, and leaf pH. Leaf thickness (Th) was measured on the lamina, while avoiding the vein using Digimatic Thickness Gauge (6 digits) (Model 547 Mitutoyo Cooperation, Kawasaki, Japan).

Specific leaf area (SLA) is the ratio of leaf area per dry mass. Leaf area was measured using the ImageJ software (Rasband, W.S., ImageJ, U. S. National Institutes of Health, Bethesda, Maryland, USA), after which the same leaf was oven dried at 60 °C for 48 hours and weighted using an analytical balance with a precision of three digits (Precisa Model XT 620M, Precisa). Chlorophyll content (SPAD) was measured at five spots on the leaf blade and averaged to obtain a single value per leaf using a chlorophyll meter (SPAD-502, Konica Minolta Sensing Europe, London, UK). Maximum quantum yield and performance index were measured by a Chlorophyll fluorescence meter (Handy PEA, Hansatech, Norfolk, UK). Maximum quantum yield or Fv/Fm is a dark-adapted technique in which a clip was placed on the blade for 15–30 minutes of each leaf prior to the measurement. Leaf pH (pH) was measured by weighting 5 grams of freshly ground leaf sample which was mixed with 40 ml of deionized water (ratio 1:8) and filtered prior to being measured with a handheld pH meter (Model PCSTestr 35, Eutech Instruments Pte Ltd, Singapore). The *t-test* statistics was used to differentiate between the mean physiology of insect and non-insect leaves at a level of 95%.

### Mitragynine quantification

Kratom leaf samples were air-dried, ground, and sieved through a mesh of size <0.5 mm. Fifty mg of ground samples were weighed into a 15 mL falcon tube, soaked in 5 mL methanol, and then sonicated (Elmasonic S, Elmasonic, Singen, Germany) for 10 minutes to be incubated overnight. The mixture was sonicated again before being centrifuged (Centrifuge 5810 R, Eppendorf, Hamburg, German) at 25^0^C and 4,500 rpm for 5 minutes. After centrifuging, the supernatant was diluted with methanol (in a ratio of 1:10) and filtered using a 0.22-μm polytetrafluoroethylene (PTFE) syringe filter into a High-performance liquid chromatography (HPLC) vial and injected for analysis.

Mitragynine accumulation was quantified through a Agilent 1260 Liquid Chromatograph (Agilent Technologies, CA, USA) combined with Inertsil ODS-3 HPLC Column, 5 µm, 150 x 4.6 mm. The HPLC system was run at a wavelength of 226 nm (4 nm bandwidth) at a column temperature of 27^0^C. For the quantification of mitragynine, mitragynine primary standard was purchased with a purity ≥ 95%, from Chromadex, Longmont, USA. The quantity of samples and standards injected into the HPLC was kept at 10 µL. The flow rate of the whole process was maintained at 1 mL/min. The mobile phase consisted of solvent A (Aqueous with 20 mM ammonium formate pH 6) and solvent B (Acetonitrile), using the gradient condition program (Table 1). Methodology related to the quantification are detailed in the supplementary file named “*S1 File*”.

**Table 1:** Gradient condition program for the mobile phase to separate mitragynine.

Time (min)	%Mobile phase A	%Mobile phase B
0	90	10
3	90	10
13	30	70
16	10	90
19	10	90
20	90	10

### Leaf metabolomic analysis

Fresh leaves of kratom were ground to a powder using liquid nitrogen with a mortar and pestle. Approximately 100 mg of the powder was placed in a 15-mL falcon tube, soaked in 5-mL of ice-cold methanol, vortexed and incubated overnight in a wrist action shaker (Rotamax 120, Heidolph, Schwabach, Germany) at 20^0^C. The soaked sample was centrifuged (Centrifuge 5810 R, Eppendorf, Hamburg, German), at 4^0^C and 6,000 rpm for 10 minutes. The sample was filtered using a 0.22 µm polytetrafluoroethylene (PTFE) syringe before injected to liquid chromatography–mass spectrometry (LC-MS). The extracted solution that was not immediately used was stored at -80˚C to prevent degradation [46].

The qualitative analyses of kratom foliar leaf metabolome was performed using a Bruker Ultra-Performance Liquid Chromatography (UPLC) system combined with a Quadrupole time-of-flight compact model mass spectrometer (Bruker, Bremen, Germany). An intensity solo C18 column (100 mm ×2.1 mm, 1.8 µm; Bruker, Bremen, Germany) was utilized to perform the chromatographic separation, which was performed at 40^0^C with a gradient elution of mobile phase consisting of 2.5 mM ammonium formate buffer in water (solvent A) and 2.4 mM ammonium formate buffer in methanol (solvent B) at a flow rate 0.4 mL/minute. The injection volume of the sample was 4 µL, which was eluded at a gradient as follows: maintain 99.9% solvent A (0.0–2.1 min), decrease to 75.0% solvent A (2.1–10.0 min), reduce to 20% solvent A (10.0–12.0 min), and drop to 0.1% solvent A (12.0–21.0 min). The gradient was then increased to 99.9% of solvent A (23.0–24.0 minutes) and held at 99.9% for 2.1 minutes. A high-resolution mass spectrometry was performed in positive mode with electrospray ionization (ESI) source using the following parameters: 500 V spray voltage, 4,500 V capillary voltage, and 4.0 L/minutes of dry gas flow rate at 200^0^C.

### Secondary metabolite identification and post-processing

The processing of untargeted mass spectrometry data was done in MS-DIAL [47], with the metabolites identified using multiple well-established databases, including RESPECT, plant_metabolome_all, GNPS, MassBank, MONA, and MSMS_insilico, all of which were downloaded from the MS-DIAL website (https://systemsomicslab.github.io/compms/msdial/main.html#MSP).RESPECT and plant_metabolome_all include plant-specific secondary metabolites or SMs, while GNPS, MassBank, MONA, and MSMS_insilico spectral libraries enabled the compound identification through spectral matching and predicted fragmentation patterns. The metabolites identified and annotated had a mass similarity range between 60% and 100%, with higher similarity scores indicating a greater confidence in compound identification. Post processing, including normalization, missing data imputation, and filtering of uninformative features, was performed using the R statistical language [42] using the Bioconductor package [48].

### Statistical analysis of untargeted metabolomics

The untargeted leaf metabolomic data obtained through LC-MS was analyzed through various statistical methods available in R statistical language [42] to determine the SM profile as well as metabolites that were significant in differentiating the classes (insect and non-insect) [49]. These methods provide a comprehensive statistical framework for analyzing complex untargeted metabolomic datasets, facilitating the identification of key changes in SMs. Principal Component Analysis (PCA) was used as a dimensionality reduction technique to capture most of the overall variance in the dataset. Data were normalized via log transformation and Pareto scaling, followed by fold change analysis (|Log2FC| > 2) and t-tests with False Discovery Rate or FDR-adjusted *P-value* < 0.05, to reduce the likelihood of false positives from multiple comparisons [50].

Significant loadings from the PCA were used to determine the metabolites contributing the most to the explained variance of the first two principle components. Partial Least Squares Discriminant Analysis (PLS-DA) was then used to maximize the separation between the predefined classes. To evaluate the robustness of the model, a train test split of 70:30 percent was used to quantify the generalization ability of model. The Variable Importance in Projection (VIP) scores derived from PLS-DA identified the most influential metabolites, providing a list of potential SMs/biomarkers for further investigation [51].

Furthermore, using an orthogonal partial least squares discriminant analysis or OPLS-DA model, a cross-validated scores plot was generated, featuring the predictive component (tp) on the x axis and the orthogonal component (to) on the y axis [52]. Additional confidence ellipses using Hotelling’s T² distribution were plotted to assess the dispersion and potential outliers within the metabolomics data. Any presence of class overlap near the origin, coupled with identified outliers beyond the Hotelling’s T² ellipse, and subsequently, using an S-plot, significant metabolites were identified using VIP scores greater than one and *P-value* < 0.1. Boxplots were then used to validate the SMs isolated from PCA, PLS-DA and OPLS-DA, to determine any significant differences in the presence of the relevant metabolites. All the analyses were conducted in R statistical language [42], with PCA done in the *base* stats package, PLS-DA using the *caret* package [53], OPLS-DA using the *ropls* which is a part of the *Bioconductor* package, and the differences between the insect and non-insect profiles were tested in the *rstatix* package [54].

## Results

### Insect diversity

**Fig 3:**
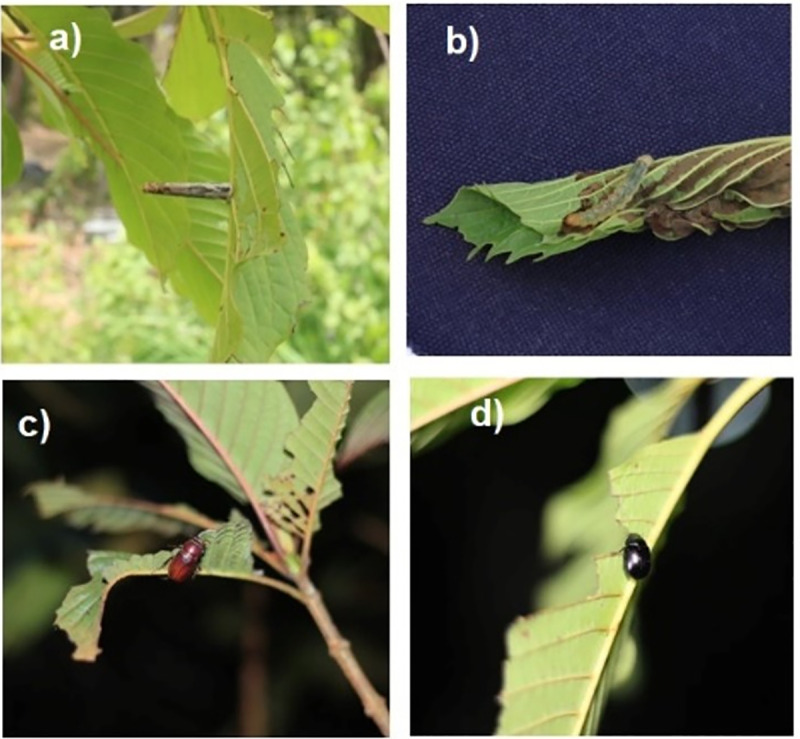
Insects identified in the kratom plantation; (a) a larva from the lepidopterous insect family, with the same morphology as LEP12, (b) a larva of *Parotis marinata* (LEP17 sample), (c) a insect beetle of *Miridiba* sp., with the same morphology as COL06, (d) a insect beetle of *Apogonia* sp., and (e) kratom stem damaged by beetle attack.

In the plantation, a total of 244 insect samples, including 187 samples of potential insect pests and 57 samples of moth samples were obtained. Twenty-eight potential insect samples were extracted for their genomic DNA, with the COI barcodes successfully amplified and sequenced. The barcodes were compared against the COI barcodes in the GenBank database, which indicated that at least 18 taxa of insects were found in the plantation (Table 2). The insects belonged to five taxa of coleopterans (beetles): *Adoretus* (Sacarabaeidae)*, Antiochrus* (Hybosoridae), *Miridiba* (Sacarabaeidae), *Sinoxylon* (Bostrichidae), and an unidentified coleopteran genus; eight taxa of lepidopterans (butterflies and moths): *Ardozyga* (Gelechiidae), *Bembina* (Erebidae), *Episparis* (Erebidae), *Homodes* (Erebidae), *Hyposidra* (Geometridae), *Parotis* (Crambidae), *Plutorectis* (Psychidae), and *Somatina* (Geometridae); and five taxa of hemipterans (true bugs): *Cixiosoma* (Cixidae), *Dryadomorpha* (Cicadellidae) and *Maiestas* (Cicadellidae), with two unidentified hemipteran genera (Fig. 3).

**Table 2:** Summary of genomic DNA analysis of the potential insect samples, including specimen ID, collecting date, stage, DNA concentration, COI barcode sequencing, and GenBank match results for insects samples collected from the kratom field in Surat Thani province.

ID	Collecting date	Stage	DNA concentration (ng/ul)	Reliable length (bp)	1^st^ GenBank match	GenBank ID	Percent identity (%)	2^nd^ GenBank match	GenBank ID	Percent identity (%)
LEP01	13-Jan-24	Larva	85.300	632	*Hyposidra talaca*	MN198978.1	100%	*Hyposidra talaca*	HM387111.1	99.20%
LEP02	13-Jan-24	Larva	75.800	623	*Bembina albinotata*	GU662685.1	97.07%	*Limbatochlamys rosthorni*	MN924362.1	92.15%
LEP03	13-Jan-24	Larva	37.400	632	*Hyposidra talaca*	MN198978.1	99.53%	*Hyposidra talaca*	HM387111.1	98%
LEP04	13-Jan-24	Larva	10.900	623	*Bembina albinotata*	GU662685.1	97.07%	*Limbatochlamys rosthorni*	MN924362.1	92.15%
LEP05	13-Jan-24	Larva	94.800	609	*Parotis marinata*	NC_087055.1	98.69%	*Parotis marinata*	HQ952930.1	98.33%
LEP06	13-Jan-24	Larva	157.40	622	*Parotis marinata*	NC_087055.1	98.55%	*Parotis marinata*	HQ952930.1	98.04%
LEP07	13-Jan-24	Pupa	194.00	605	*Parotis marinata*	NC_087055.1	98.69%	*Parotis marinata*	HQ952930.1	98.33%
LEP08	13-Jan-24	Larva	99.350	636	*Plutorectis pelloceros*	HQ923262.1	86.06%	*Pteroma pendula*	KY448246.1	85.58%
LEP10	13-Jan-24	Adult	32.500	623	*Parotis marinata*	NC_087055.1	98.72%	*Parotis marinata*	HQ952930.1	98.21%
LEP11	26-Mar-24	Adult	13.550	634	*Episparis liturata*	OQ512128.1	95.62%	*Episparis liturata*	OQ512125.1	95.62%
COL01	26-Mar-24	Adult	51.050	566	*Adoretus ovalis*	MZ836004.1	88.36%	*Adoretus hirsutus*	OQ428819.1	88.35%
COL02	26-Mar-24	Adult	22.000	609	*Antiochrus aberrans*	MW340066.1	86.22%	*Tachyporus pusillus*	KJ962073.1	86.81%
COL03	26-Mar-24	Adult	17.250	627	*Sinoxylon anale*	LC612432.1	99.17%	*Sinoxylon anale*	LT904780.1	92.52%
HEM02	26-Mar-24	Adult	33.350	634	*Hemipteran sp*	JQ344801.1	99.14%	*Cicadellidae*	KY836411.1	99.24%
HEM03	26-Mar-24	Adult	80.000	651	*Cixiosoma*	OR721858.1	83.33%	*Cixiosoma*	OR721855.1	83.36%
LEP12	26-Mar-24	Larva	254.90	383	*Ardozyga obeliscota*	JN270847.1	91.64%	*Scythris*	MH417395.1	91.38%
LEP14	26-Mar-24	Larva	59.250	629	*Somatina*	KF388689.1	97.90%	*Somatina*	KF392122.1	97.74%
LEP15	26-Mar-24	Larva	33.150	623	*Bembina albinotata*	GU662685.1	97.07%	*Limbatochlamys rosthorni*	MN924362.1	92.15%
HEM04	26-Mar-24	Adult	17.150	640	Alydidae	OM323150.1	99.53%	*Riptortus serripes*	HQ958376.1	98.89%
HEM05	26-Mar-24	Adult	209.10	623	*Dryadomorpha*	KX437736.1	99.84%	Cicadellidae	MW429216.1	93.21%
Lep16	28-Mar-24	Pupa	4.4000	632	*Homodes crocea*	HM877094.1	95.35%	*Homodes iomolybda*	MT256676.1	95.18%
COL06	15-Jun-24	Adult	54.200	568	*Miridiba saigonensis*	OR753652.1	93.47%	*Phyllophaga ephilida*	EU156680.1	85.89%
COL08	15-Jun-24	Adult	47.600	485	Coleoptera sp. XSBNHap_219	JQ344775.1	99.79%	*Adoretus tenuimaculatus*	KC510117.1	91.98%
LEP17	15-Jun-24	Larva	137.40	611	*Parotis marinata*	NC_087055.1	98.69%	*Parotis marinata*	HQ952930.1	98.34%
LEP18	15-Jun-24	Larva	331.25	634	*Episparis liturata*	OQ512128.1	95.79%	*Episparis liturata*	OQ512125.1	95.79%
LEP19	15-Jun-24	Larva	124.95	632	*Somatina*	KF388689.1	97.91%	*Somatina*	KF392122.1	97.75%
HEM06	15-Jun-24	Adult	52.250	634	*Cixiosoma*	OR721858.1	83.87%	*Kirbyana lini*	KF818476.1	82.97%
HEM07	15-Jun-24	Adult	32.700	622	*Maiestas xanthocephala*	MT998319.1	90.56%	Cicadellidae sp. 6	MW429222.1	87.50%

### Quantification of mitragynine

**Fig 4:**
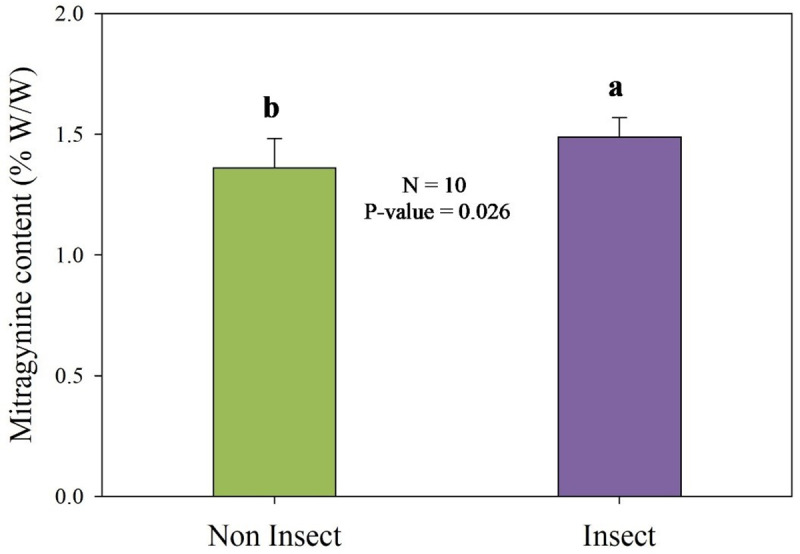
Mitragynine content found in kratom leaves collected from undamaged (Non-Insect) and insect-damaged (Insect) leaves. The *P-value* represents the result of a statistical test using the t-test at a 95% confidence level.

Leaf morphology and physiology of insect and non-insect leaves was not statistically different as indicated in Table 3. The only difference was quantified in insect-damaged leaves that exhibited significantly higher acidity (lower leaf pH) compared to undamaged leaves, as indicated in Table 4. The results of this comparative analysis are illustrated in Fig 4. The mitragynine content was found to be significantly higher in insect-damaged leaves compared to undamaged leaves (P-value <0.05). The concentrations ranged between 1.25% and 1.58% by weight, which is within the normal range.

**Table 3:** Morphological and physiological characteristics of insect-infested kratom leaves and healthy leaves in Nampu Subdistrict, Ban Na San District, Surat Thani Province. The statistical difference (*P-value*) is obtained from the t-test statistical test, where a value greater than 0.05 indicates no statistically significant difference between the two groups, while ^***^ indicates a significant difference. The superscripted letters indicate any differences in mean comparison.

Leaf characteristics	insect-infested leave	Healthy leaves	Degrees of freedom	t-value	*P-value*
Leaf Thickness (mm)	0.16 ± 0.03	0.15 ± 0.02	98	0.801	0.424
Specific Leaf Area (SLA) (cm2 g-1)	208.40 ± 43.74	196.58 ± 40.1	98	1.408	0.162
Chlorophyll Content (SPAD)	31.77 ± 3.99	31.06 ± 4.06	98	0.874	0.384
Quantum Yield (Fv/Fm) (Unitless)	0.82 ± 0.01	0.82 ± 0.01	98	0.765	0.446
Performance index (PI) (Unitless)	2.43 ± 0.74	2.28 ± 0.73	98	1.010	0.315
Leaf pH (pH) (Unitless)	**4.47 ± 0.08** ^**a**^	**4.33 ± 0.05** ^**b**^	98	**10.408**	<0.0001***

**Table 4:** Summary of the number of SMs, with those significantly loading the first two principle axes (PC) of PCA also indicated.

Total SMs identified	Significant number of SMs	SMs identified as UP [PC1/PC2]	SMs identified as DOWN [PC1/PC2]	SMs with no change [PC1/PC2]
139	62	36/17	0/18	26/27

### Quality control, screening and differential presence of metabolites

**Fig 5:**
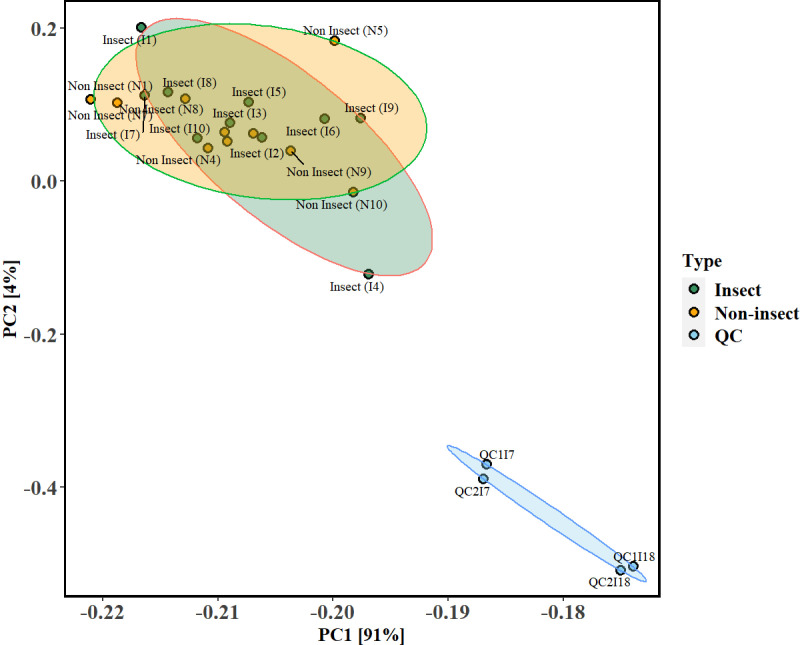
PCA score plots as obtained from the LC-MS data for *M. speciosa* or kratom leaves characterized by insect and non-insect groups for the positive ion mode.

The metabolite profiling was characterized based on the insect and non-insect groups, followed by qualitative analysis. Quality control (QC) and data integrity checks indicated no missing values for untargeted metabolomics (the interested reader can check S1 Table). The QC samples formed a distinct cluster, indicating good reproducibility and stability in the QC data. The PCA plot for the metabolic profiles of both groups and QC samples is shown in Fig 5, with all samples clustering together and the first two principal components explaining around 95% of the total variance. The post-processing resulted in 139 metabolites (S2 Table) that constituted the metabolomics profile of the two groups of kratom.

**Fig 6:**
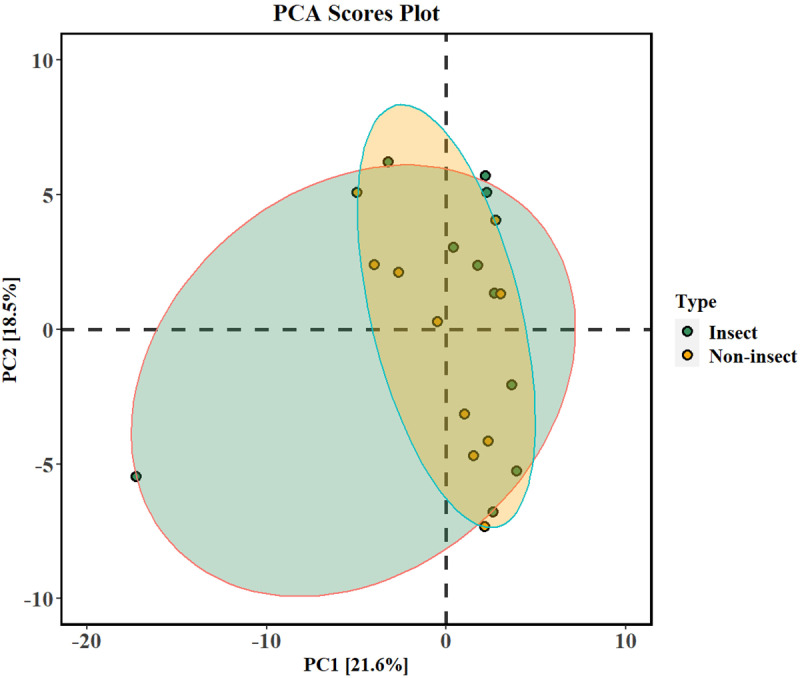
The PCA scores plot characterizing the separation of insect (green) and non-insect (orange) samples based on the first two principal components, PC1 and PC2. The variance explained by the first two principle components is indicated in the axis labels.

Principal Component Analysis (PCA) scores plot (Fig 6) derived from untargeted metabolomics data, showing the clustering of insect and non-insect samples based on their comprehensive metabolite profiles. The first two principal components (PC1 and PC2) explain a combined 40.1% of the variance. Ellipses represent the 95% confidence intervals for each group, illustrating considerable similarity in the metabolite profiles of the insect and non-insect groups, with greater variability within the non-insect group. From the 139 metabolites, the loadings plot indicated that 62 SMs significantly loaded the first two principle axes of the PCA (see Table 4).

**Fig 7:**
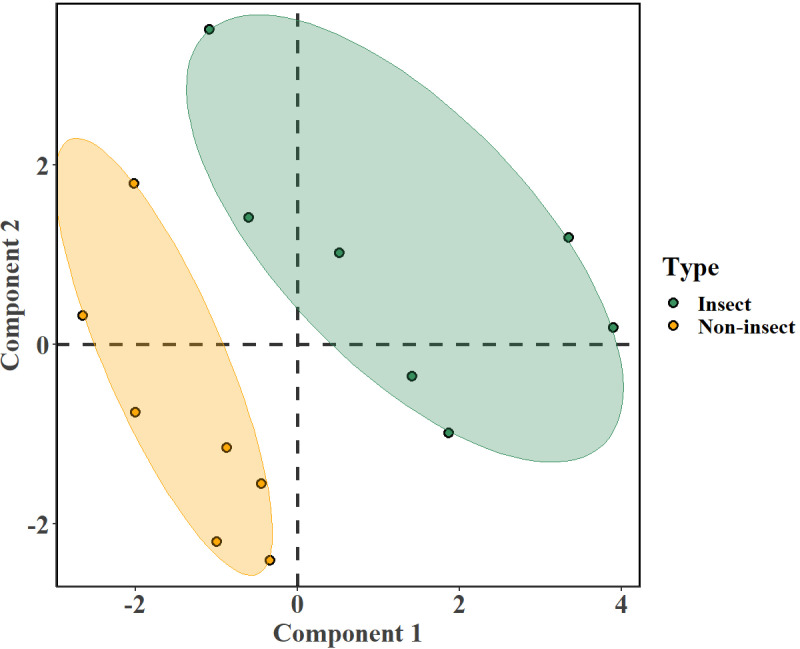
The PLS-DA plot characterizing the separation between insect (green) and non-insect (orange) samples using two latent variables, Component 1 and Component 2.

**Fig 8:**
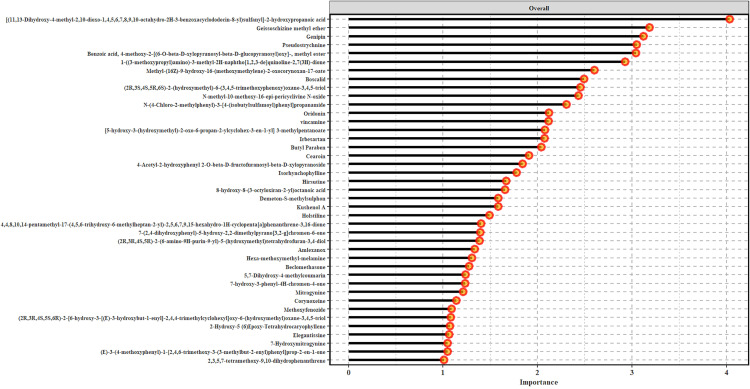
The VIP scores identifying SMs that significantly contributed to differentiating between the insect and non-insect samples as determined by the PLS-DA model. Chemical names are listed in supplementary Table S3.

PLS-DA was used to investigate the separation between insect and non-insect samples under positive ion mode, whose first two latent variables (Component 1 and Component 2) resulted in a pronounced separation between the two groups (Fig 7). The leaf samples that had visible insect damage (green) formed a well-defined cluster with minimal overlap with non-insect samples (orange). The model validation indicated a classification accuracy of around 70%, further demonstrating its effectiveness in distinguishing between the two groups. A clear discrimination through PLS-DA indicated that the samples from the two groups, with the SM features most significant (VIP scores greater than 1) in discriminating between the insect and non-insect groups determined through the VIP plot in Fig 8. The VIP scores and ontologies of SMs are listed in the supplementary S3 Table.

Fig 4 contains the VIP scores of metabolites greater than one, with the length of the horizontal bar corresponding to the VIP score of respective metabolite. Out of the 139 metabolites 46 unique were found to have VIP score above one, with SMs such as [(11,13)-Dihydroxy-4-methyl-2,10-dioxo-1,4,5,6,7,8,9,10-octahydro-3H-3-benzazocyclodecin-8-yl]propanoic acid and geissoschizine methyl ether having the highest VIP scores and significantly contributing to the discrimination between the two sample groups. Several well-known kratom metabolites also significantly contributed to discriminating between insect and non-insect sample groups which included holstiline, hirsutine, 7-hydroxymitragynine, corynoxeine, and mitragynine, with VIP scores ranging between 1.2 (7-hydroxymitragynine) to 1.8 (hirsutine). The analysis identified differential metabolites that significantly contributed to this categorization, with a closer look at the SMs listed in the VIP indicating that 27 metabolites that included SMs previously reported such as corynoxine, isorhynchophylline, holstiline, and 7-hydroxymitragynine also significantly loading the PCA axes.

**Fig 9:**
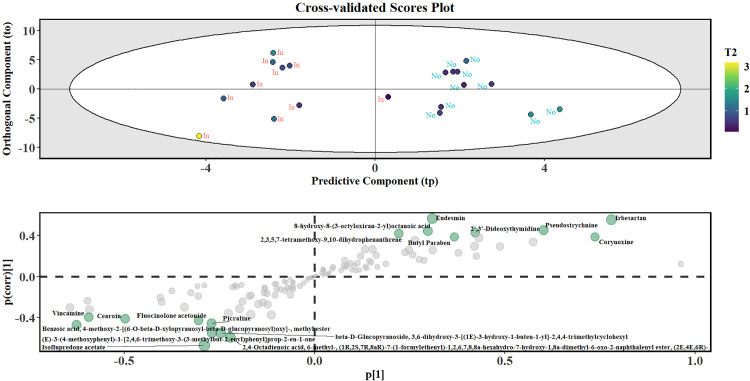
Cross-validated scores plot using OPLS-DA scatter plot (top panel) to determine the class separation between the insect and non-insect groups. The points are labeled based on class membership into “In” and “No”, with the x and y-axes representing the predictive (tp) and orthogonal (to) components and the ellipse representing the 99% confidence intervals as determined by the Hotelling’s T² distribution. The scatterplot between the covariance and correlation or the S-plot is shown in the bottom panel, highlighting key metabolites contributing to group discrimination, based on *P-value* < 0.1 and VIP > 1.

Fig 9 presents the results of cross-validated OPLS-DA (top panel) and the respective S-plot (bottom panel) based on the insect-damaged and non-insect leaf samples, Points inside this elliptical region are within the 99% confidence limit for T^2^ Hotelling’s test. Distinct clusters with minimal overlap were obtained between non-insect and insect samples. The colored gradient on the points corresponds to their T² statistic, indicative of the measure of how far each point was from the centroid of the class distribution. Darker colored points were closer to while brighter colors were further from the centroid. The S-plot (right panel) complements this by displaying the covariance and correlation of metabolites with the predictive component. Key metabolites, such as 2,3,5,7-tetramethoxy-9,10-dihydrophenanthrene, 8-hydroxy-8-(3-octyloxiran-2-yl) octanoic acid, and monopalmitin, were identified as having high importance and strong correlation with the group separation, as they were located on the extremes of the plot.

**Fig 10:**
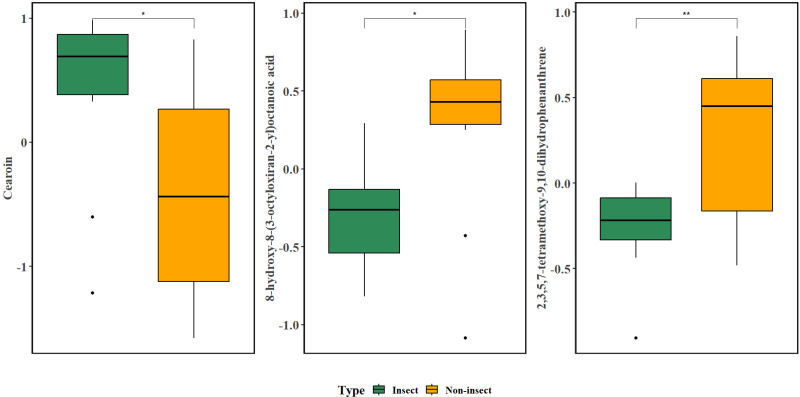
Box plots comparing the relative abundance of three metabolites between “Insect” (green boxes) and “Non-insect” (orange boxes) groups, with the metabolite indicated on the y-axis. Statistical significance is indicated by * (*P-value* < 0.0) and ** (*P-value* < 0.01).

Further analysis of the relative presence of the SMs was done using box-plots in Fig 10, to highlight any significant differences in their presence based on the observed interaction with insects. The analysis was run only on the metabolites that significantly loaded the PCA axes, while having a VIP score greater than 1. The figure presents box plots of three specific metabolites with significantly different relative abundance between insect-damaged (green) and non-insect (orange) samples. These include cearoin, 8-hydroxy-8-(3-octyloxiran-2-yl) octanoic acid, and 2,3,5,7-tetramethoxy-9,10-dihydrophenanthrene. The insect-damaged samples exhibited significantly higher relative abundance of cearoin compared to the non-insect samples, at a significance level of p < 0.05, with the non-insect samples having greater variability, and a wider interquartile range (IQR). Conversely, the presence of 8-hydroxy-8-(3-octyloxiran-2-yl) octanoic acid and 2,3,5,7-tetramethoxy-9,10-dihydrophenanthrene was significantly higher in the non-insect samples, with 2,3,5,7-tetramethoxy-9,10-dihydrophenanthrene showing a highly significant presence (*P-value* < 0.01). The non-insect samples had a significantly higher presence and narrower IQR for 8-hydroxy-8-(3-octyloxiran-2-yl) octanoic acid, indicating a lower variability in its presence relative to the insect group.

## Discussion

The interaction resulting from biotic stressors related to herbivory can play a crucial role in influencing the presence of plant SMs, which are essential for plant defense against herbivory. In the present study, untargeted metabolomics, quantification of mitragynine as well as correspondence with physiological traits were used to identify the leaf metabolome through LCMS and the identification of metabolites whose presence was significantly different between insect and non-insect samples.

To our knowledge till date, this is the first report of insect species that damage kratom leaves. Most of the insects found in this study were defoliators and leaf miners with chewing damage to the leaves. It is likely to be that accumulation of chemicals especially alkaloids (such as mitragynine quantified in the present study), which result in a bitter taste, from an increase in accumulation after insect damage as a repellence or defense mechanism to prevent further damage from insect. Recently, Ruddit et al. [55] verified the potential of kratom extracts in controlling insects through a crude extract by hexane. The authors reported its effectiveness against adult of sweet potato weevil (*Cylas formicarius*) as it had insecticidal properties, killing ability, repellence, and inhibition of oviposition activities to insect.

The untargeted metabolomic analysis initially involved the use of PCA, which was not able to properly separate the insect from the non-insect samples, with 62 metabolites from various ontologies significantly loading the first two principle axes. Previous studies have used PCA in metabolomics for dimensionality reduction and as a first step to characterizing samples and determining important metabolites through significant loadings. For instance, Liess et al. [56] used PCA on metabolomics data to differentiate the insect resistant *Dendranthema grandiflora* (chrysanthemum) cultivars from the susceptible ones, with the PCA not able to clearly distinguish the two groups. However, loadings indicated that the resistant samples exhibited higher levels of specific acids, while susceptible samples had elevated levels of malic acid and apigenin glycoside. On the other hand, Gu et al. [57] observed a clear separation between insect and non-insect samples of rice using PCA, capturing the key characteristics of all samples.

The use of PLSDA resulted in SMs from various ontologies being identified as significant discriminators between insect and non-insect metabolomes with reported therapeutic benefits (see S3 Table). These included anti-inflammatory activity of 7-hydroxycoumarins [58], which alongside p-hydroxybenzoic acid alkyl esters, also exhibit antimicrobial properties [59]. Additionally, iridoids and their derivatives present in the samples are known for their anti-inflammatory and anticancer effects, particularly in traditional medicine [60]. Other notable ontologies included isoflavones, recognized for their estrogenic properties that can reduce the risk of hormone-related cancers [61] and benzophenones for their UV-protective properties [62]. Furthermore, alkaloids such as strychnos and corynanthean-type alkaloids, that include mitragynine and 7-hydroxymitragynine, are of particular interest due to their psychoactive and analgesic properties [63].

A significantly higher presence of cearoin was detected in leaf samples attacked by insects and belongs to the family of benzophenones (see S3 Table). This compound is a neo-flavonoid [64] has also been previously isolated in several *Dalbergia* species such as *D. cearensis* Ducke, *D. miscolobium* Benth, and *D. odorifera* has been reported to have antiallergic properties [65]. A recent study by Bastola et al. [66] also suggested that it could have therapeutic potential in treating neuroblastoma by inducing autophagy and apoptosis of the cancerous cells.

8-hydroxy-8-(3-octyloxiran-2-yl) octanoic acid is a SM from the ontology of lineolic acids and derivatives. Its presence was significantly lower in the insect samples and could be attributed to this family of SMs being precursors to the production of phytohormone jasmonic acid or JA, which has been reported to have a defensive role against biotic stressors [67]. Although JA was detected in the preprocessed leaf samples (see S1 Table), the final metabolomics list did not feature JA as its signal was below the noise floor (see S2 Table). Previously, War et al. [68] had reported that wounding and chewing by herbivory or insects converts linolenic acid into JA. This spending of the SMs such as 8-hydroxy-8-(3-octyloxiran-2-yl) octanoic acid might result in the significantly lower presence observed in the insect leaf samples in the present study.

A significantly higher presence of 2,3,5,7-tetramethoxy-9,10-dihydrophenanthrene was observed in the non-insect group and is a positional isomer of 2,4,6,7-tetramethoxy-9,10-dihydrophenanthrene reported previously by Majumder et al. [69]. This compound belongs to a subclass of phenanthrenes, which have garnered interest due to their diverse biological activities that include cytotoxic, antimicrobial, anti-inflammatory, anticancer, and antioxidant effects [70]. Naturally occurring phenanthrenes have been reported in the Orchidaceae family [71] as well as Juncaceae and Euphorbiaceae [70].

The observed mitragynine accumulation levels in insect-damaged leaves are consistent with findings presented in previous studies. For example, Leksungnoen et al. [40] and [72] reported mitragynine levels between 0.75% and 2.66% in kratom leaves from Thailand, suggesting that mitragynine production in kratom trees aged 1–2 years can reach levels comparable to those in fully matured trees,. Mitragynine, as an SM, depends on energy from primary metabolites produced through photosynthesis, which are primarily allocated for growth. Suboptimal growth can affect the production of SMs, but despite potential limitations, we observed a significant difference in the accumulation of mitragynine between insect-damaged and undamaged leaves. This supports the hypothesis that insect damage can stimulate a higher alkaloid production, particularly of bitter compounds like mitragynine, to deter further insect predation.

When plants experience biotic stress, such as insect infestation, there is often a marked increase in the production of alkaloids as a part of broader strategy to prevent further damage. The production of alkaloids in response to herbivory has been well-documented across various plant species. Previously, Baldwin and Ohnmeiss [73] reported that leaf damage to the Nicotiana species native to North America attacked by herbivores resulted in 1.5- to 5-fold increases in the accumulation of primary leaf alkaloids. Vilariño et al. [74] analyzed the relationship between insect herbivory and elevated alkaloid-induced bitterness in *Lupinus albus* (Rumbo) leaves and concluded that both the sweet and bitter varieties exhibited a significant increase in alkaloid concentration. A similar observation was made by Frick et al. [75], who concluded that the level of quinolizidine alkaloids was higher in bitter narrow-leafed lupin plants.

Insect attacks can be significantly influenced by foliar pH [76], affecting both insect feeding behavior and plant responses. Researches indicate that variations in leaf pH can alter the interactions between plants and insects, impacting their feeding efficiency and preferences. It has been reported that the presence of organic acids and carbon-rich SMs can lower leaf pH [77]. Leaf pH is also strongly linked to digestibility, making it a key predictor of herbivore palatability, by making the leaves taste acidic, lowering any subsequent damage. Studies have also reported a strong correlation between lower pH and increased total flavonoid (TF) content in leaves, indicating that the surrounding media’s pH significantly influences the accumulation of SMs, enhancing flavonoid production [78].

The increased acidity observed in insect-damaged leaves may contribute to the enhanced bitterness linked to higher alkaloid content, which may affect the taste or toxicity of the leaves to insects [79]. This phenomenon is consistent with findings in other plant systems, such as the increased production of antimicrobial benzophenanthridine alkaloids in opium due to temporary cellular acidification [80]. Higher accumulation of mitragynine in insect-damaged leaves thus suggest a defensive response, where increased leaf acidity (lower foliar pH) plays a critical role in enhancing this response.

The study concludes that insect damage triggers a higher accumulation of mitragynine in kratom, enhancing its natural defenses without the need for insecticides. This finding suggests that allowing controlled insect herbivory could be an economical and organic strategy to boost plant resilience. By leveraging the plant’s innate response, farmers can reduce chemical inputs, leading to more sustainable agricultural practices. This approach not only promotes ecological balance but also offers a cost-effective alternative to conventional insect control methods.

## Conclusion

This study investigated the relationship between insect preference and the probable biochemical responses in kratom leaves, focusing on insect diversity, untargeted metabolomics, and the accumulation levels of mitragynine. The biotic pressures faced by kratom plants was highlighted by the presence of multiple herbivore species, particularly beetles. Through untargeted metabolomics, three metabolites were found to be notably different between insect-damaged and non-damaged leaves, with damaged leaves accumulating higher levels of mitragynine. Furthermore, a correspondence was observed between increased alkaloid production and heightened leaf acidity in insect-damaged leaves. This observation demonstrates that insect herbivory can increase the production of defensive and bitter compounds like alkaloids, with corresponding increase in acidity deterring herbivory. Such dynamics between insect diversity, metabolomic changes, and alkaloid accumulation underscores the complex interplay between kratom plants and their herbivores.

## Supporting information

S1 Table List of metabolites obtained from MS DIAL. The file includes an inexhaustive list of metadata such as retention times, relative mass, metabolite name as annotated using several metabolite databases, metabolite similarity in terms of mass, blank, QCs, and metabolite features per sample (10 replicates of insect and non-insect leaves).
(CSV)

S2 Table List of post processed metabolites. The file includes an inexhaustive list of metadata such as retention times, relative mass, metabolite name as annotated using several metabolite databases, metabolite similarity in terms of mass, blank, QCs, and metabolite features per sample (10 replicates of insect and non-insect leaves).
(CSV)

S3 Table List of metabolites deemed as significant discriminators (VIP>1) between insect and non-insect metabolome as per the PLSDA. (CSV)

S1 File This file outlines the quantification process of mitragynine in kratom samples. (DOCX)
