# Supplementary material for: Higher accumulation of mitragynine in Mitragyna speciosa (kratom) leaves affected by insect attack
Source: PLoS One. 2025 Apr 29;20(4):e0320941. doi: 10.1371/journal.pone.0320941 (PMC12040156; doi:10.1371/journal.pone.0320941)
Supplement: S1 File — (DOCX) [file pone.0320941.s004.docx]

**Mitragynine quantification**

The kratom leaf samples were air-dried, ground, and filtered through a mesh of size <0.5 mm. Fifty mg of the ground samples were weighed into a 15 mL falcon tube and soaked in 5 mL methanol and sonicated for 10 minutes (Elmasonic S, Elmasonic, Singen, Germany) and incubated overnight. The mixture was sonicated before being centrifuged (Centrifuge 5810 R, Eppendorf, Hamburg, German) at 25 ̊C and 4,500 rpm for 5 minutes. After centrifuging, the supernatant was diluted with methanol (at a ratio of 1:10). A dilution sample was filtered using a 0.22-μm polytetrafluoroethylene (PTFE) syringe filter into an HPLC vial and injected for HPLC analysis.

Mitragynine primary standard (purity ≥ 95%, Chromadex, Longmont, USA) was prepared for constructing the calibration curve. One mg/mL of the stock standard solution was prepared as the working solution by diluting 0.1 mg/mL of the mitragynine primary standard with methanol. Dilution of working solution with methanol were done to have eight concentration points that were set at 0.001, 0.005, 0.008, 0.010, 0.012, 0.015, 0.018, and 0.020 mg/mL of mitragynine. All solutions that were not used immediately were stored at -20˚C after preparation.

Quantification of mitragynine in the leaves of kratom was done by an Agilent 1260 Liquid Chromatograph (Agilent Technologies, USA) combined with the Inertsil ODS-3 HPLC Column, 5 µm, 150 x 4.6 mm. The HPLC system was run at a wavelength of 226 nm (4 nm bandwidth) and a column temperature 27˚C. Ten µL of all samples and standards was injected into the HPLC instrument. The whole procession was conducted at a flow rate of 1 mL/min. The mobile phase consisted of solvent A (Aqueous with 20 mM ammonium formate pH 6) and solvent B (Acetonitrile) using the gradient condition program (listed in Table 1).

**Table 1.** Gradient condition program of the mobile phase for separation of mitragynine.

| Time (min) | %Mobile phase A | %Mobile phase B |
| --- | --- | --- |
| 0 | 90 | 10 |
| 3 | 90 | 10 |
| 13 | 30 | 70 |
| 16 | 10 | 90 |
| 19 | 10 | 90 |
| 20 | 90 | 10 |

The HPLC conditions used in this study had the presence of a mitragynine chromatogram around a retention time of 14.26 minutes (Fig 1). The specificity, indicated by the peak purity, was measured at 0.99 (Fig 2). Linearity, as determined through the mitagynine reference standard, exhibited a linear regression coefficient (R^2^) of 0.9993 (Fig 3) across a concentration range of 0.001 to 0.020 mg/mL. Other parameters assessing the method's effectiveness, including resolution, theoretical plate count and tailing factor, were found to be satisfactory. A signal-to-noise ratio of 10 was used for mitragynine, with a concentration of 0.027 mg/mL. Setting the lower limit of detection at 0.001 mg/mL resulted in an excellent precision. The accuracy, precision and %recovery data are shown in the Table 2.

a.
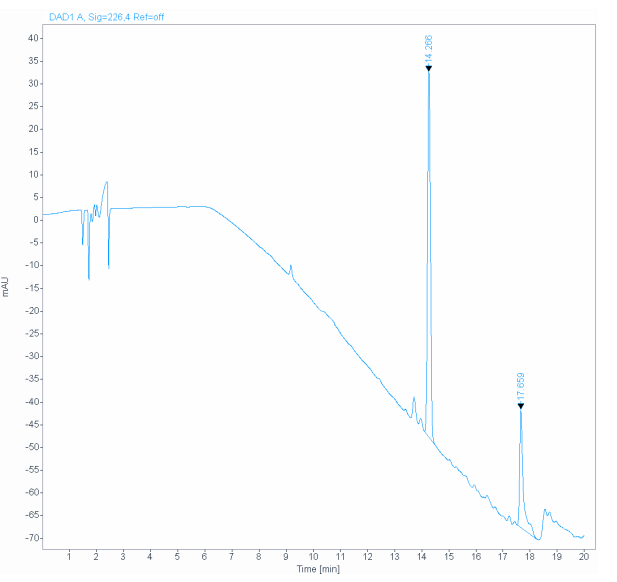
 b.
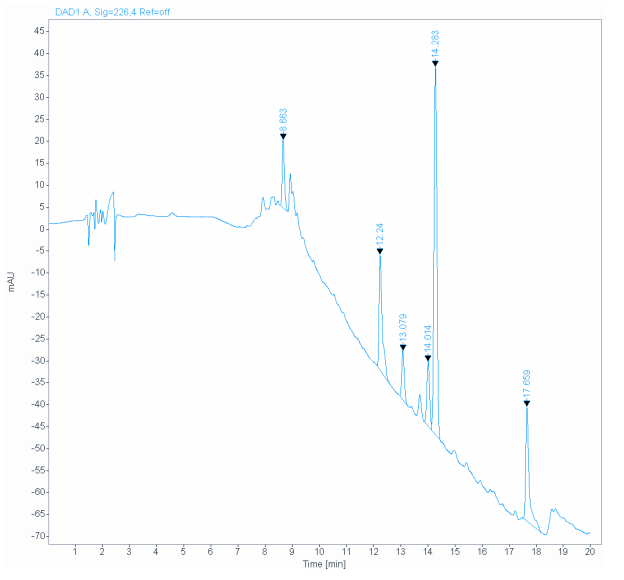


c.

**Fig 1.** Chromatogram of mitragynine at 14.26 minutes in each source
a. mitragynine reference standard 0.008 mg/mL b. kratom’s leaf extraction c. blank


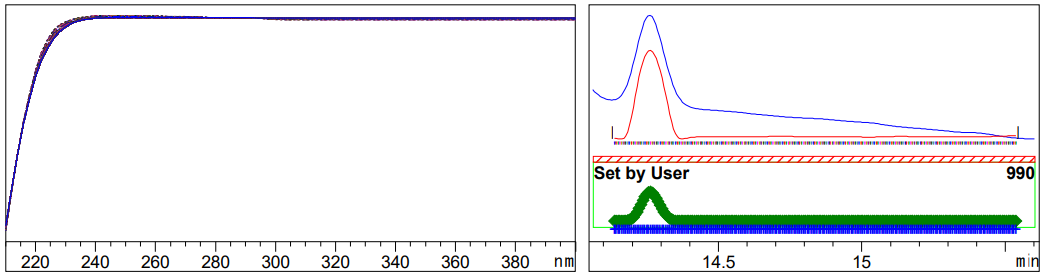


**Fig 2.** Peak purity of mitragynine from kratom’s leaf extraction

**Fig 3.** The standard calibration curve and linear equation of mitragynine which concentration range between 0.001 to 0.020 mg/mL

**Table 2.** Accuracy and precision of the analysis of mitragynine

| **Name** | **Intra-day (n=9)** | | **Inter-day (n=9)** | |
| --- | --- | --- | --- | --- |
|  | **% Recovery** | **% RSD** | **% Recovery** | **% RSD** |
| Low  (0.005 mg/mL) | 93.23 | 2.09 | 104.01 | 1.71 |
| Medium  (0.010 mg/mL) | 95.46 | 1.54 | 105.28 | 2.96 |
| High  (0.0015 mg/mL) | 96.89 | 1.88 | 108.13 | 1.35 |
